# PREVALENCE OF MALNUTRITION, ACCORDING TO THE GLIM CRITERIA, IN PATIENTS WHO ARE THE CANDIDATES FOR GASTROINTESTINAL TRACT SURGERY

**DOI:** 10.1590/0102-672020210002e1663

**Published:** 2022-06-24

**Authors:** Maurício Luann Dantas dos SANTOS, Luana de Oliveira LEITE, Isolda Carneiro Freitas LAGES

**Affiliations:** 1 University of the State of Bahia, Department of Life Sciences - Campus I - Salvador - Bahia - Brazil;; 2 Roberto Santos General Hospital, Nutrition Unit - Salvador - Bahia - Brazil

**Keywords:** Nutritional Status, Malnutrition, General Surgery, Estado nutricional, Desnutrição, Cirurgia Geral

## Abstract

Surgical patients constitute a group of individuals who are commonly underdiagnosed and undertreated, where nutritional impairment can be either a preexisting finding or a result of the hypercatabolic and hypermetabolic state.

**OBJECTIVE:**

The purpose of this study was to assess the prevalence of malnutrition, according to the GLIM criteria, and its association with clinical and nutritional factors, in individuals admitted to a surgical unit of a general hospital.

**METHODS:**

A cross-sectional, retrospective study was conducted, involving patients in the preoperative period due to gastrointestinal diseases. Demographic, clinical, and nutritional data were collected from adult and elderly patients admitted to a surgical unit between March and December 2019. Nutritional risk was assessed using the Nutritional Risk Screening tool (NRS-2002). The prevalence of malnutrition was found using the GLIM criteria. Binary logistic regression modeling was performed to determine the association between the diagnosis of malnutrition using the GLIM method and clinical and nutritional variables.

**RESULTS:**

The majority of the sample presented nutritional risk (50.2%) according to the NRS-2002. The prevalence of malnutrition according to the GLIM criteria was 32.3%, with severe malnutrition being predominant (21.2%) in all age groups. There was an association between malnutrition and nutritional risk detected by the NRS-2002 (OR: 5.791; 95%CI 3.201-10.478). There was a predominance of patients undergoing cancer surgery (64%) and these patients were more likely to be diagnosed with malnutrition (OR: 2.068; 95%CI: 1.161-3.683), after statistical adjustment.

**CONCLUSION:**

An important prevalence of nutritional risk assessed by the NRS-2002 and of malnutrition assessed by the GLIM method was identified, especially in its severe form. In addition, preoperative patients with nutritional risk, as detected using the NRS-2002 nutritional screening tool, and candidates for oncologic surgery are more likely to be diagnosed as malnourished using the GLIM criteria.

## RESUMO - RACIONAL

Os pacientes cirúrgicos constituem um grupo de indivíduos comumente subdiagnosticado e subtratado, onde o comprometimento nutricional pode ser tanto um achado preexistente quanto decorrente do estado hipercatabólico e hipermetabólico.

**OBJETIVO:**

Avaliar a prevalência de desnutrição, de acordo com os critérios GLIM (*Global Leadership Initiative on Malnutrition*), e sua associação com fatores clínicos e nutricionais, em indivíduos internados em uma unidade cirúrgica de um hospital geral.

**MÉTODOS:**

Estudo transversal, retrospectivo, envolvendo pacientes no pré-operatório por doenças gastrointestinais. Foram coletados dados demográficos, clínicos e nutricionais de pacientes adultos e idosos admitidos entre março e dezembro de 2019 em uma unidade cirúrgica. O risco nutricional foi avaliado pela ferramenta *Nutritional Risk Screening* (NRS 2002). A prevalência de desnutrição foi encontrada a partir dos critérios GLIM. Modelos de regressão logística binária foram realizados para determinar a associação entre o diagnóstico de desnutrição pelo método GLIM e variáveis clínicas e nutricionais.

**RESULTADOS:**

A maior parte da amostra apresentou risco nutricional (50,2%) conforme NRS 2002. A prevalência de desnutrição pelos critérios GLIM foi 32,3%, sendo a desnutrição grave predominante (21,2%) em todas as faixas etárias. Verificou-se associação entre a desnutrição e o risco nutricional detectado pela NRS-2002 (OR: 5,791; IC5% 3,201-10,478). Observou-se predominância de pacientes candidatos a cirurgias oncológicas (64%) e estes apresentaram maiores chances de receber o diagnóstico de desnutrição (OR: 2,068; IC95%: 1,161-3,683), após ajuste estatístico.

**CONCLUSÃO:**

Foi Identificado importante prevalência de risco nutricional avaliado pela NRS-2002 e de desnutrição pelo método GLIM, principalmente na forma grave. Além disso, os pacientes no pré-operatório com risco nutricional, detectado por meio da ferramenta de triagem nutricional NRS 2002, e aqueles candidatos à cirurgia oncológica têm mais chances de serem diagnosticados como desnutridos pelos critérios GLIM.

## INTRODUCTION

Malnutrition can be defined as “the state resulting from nutrient deficiency that can cause changes in body composition, functionality and mental status with a loss in the clinical outcome”^26^. It is a multifactorial disease that is quite prevalent throughout the world and is responsible for high mortality rates, worsening of the immune response, difficulty in the healing process, and increased risk of surgical and infectious complications^17^ ^,^ ^5^ ^,^ ^13^ ^,^ ^27^.

In clinical practice, the term “malnutrition” has been used mainly to characterize the state of nutritional deficiency^6^ whose prevalence varies between 20% and 80% of patients in the hospital environment^11^ ^,^ ^16^. It is noteworthy that about 70% of malnourished patients worsen in their nutritional status during their stay in hospitals^25^. In the case of patients with intra-abdominal diseases, for whom surgery is the only possible curative treatment option, attention to the diagnosis of malnutrition is essential^22^. Therefore, surgical patients constitute a group of individuals who are commonly underdiagnosed and undertreated, where nutritional impairment can be either a preexisting finding or a result of the hypercatabolic and hypermetabolic state presented^8^ ^,^ ^18^ ^,^ ^29^. In this population, the prevalence of malnutrition varies from 8.9% to 33.4%, depending on the evaluation method used^27^.

Although there is concern about the clinical and functional outcomes associated with malnutrition, there is a lack of consensus on the applicable diagnostic criteria. In addition to failures in the recognition and treatment of malnutrition, the multiplicity of terminologies points to both the frail setting and the need to establish a single definition^2^ ^,^ ^6^. Thus, in 2016, an international working group called the Global Leadership Initiative on Malnutrition (GLIM) was created, with the objective of establishing a method to unify the diagnosis of malnutrition ^4^. The GLIM method has five diagnostic criteria structurally divided between phenotypes and etiologies, so that the diagnosis requires the association of at least one criterion from each group.

This new proposal to unify the diagnosis of malnutrition is of fundamental importance in the early identification of compromised nutritional status, which is a predictor of worse clinical outcomes for surgical patients. However, establishing the diagnosis of malnutrition does not in itself improve the clinical evolution of the patient, since the nutritional diagnosis represents the link between nutritional assessment and intervention. Therefore, this study proposes to provide not only recent and relevant data for recognizing cases of malnutrition using a current method in surgical patients but is also important with regard to the undertaking of adequate nutritional interventions and, consequently, reduction of morbidity, mortality, and hospital costs in this population. Thus, this study aimed to evaluate the prevalence of malnutrition, according to the GLIM criteria, and its association with clinical and nutritional factors in individuals admitted to the surgical unit of a general hospital.

## METHODS

This is a cross-sectional, retrospective study, conducted from the analysis of clinical records of individuals in the preoperative period, admitted between March and December 2019 to an inpatient surgical unit of a reference General Hospital in general surgery, located in the city of Salvador, Bahia, Brazil. This surgical unit admits patients who are the candidates for surgery, predominantly for gastrointestinal diseases (oncological or otherwise), including the surgical treatment of diseases that affect the digestive system (e.g., hernias, diverticulitis, hepatic cysts, gallstones, gastrointestinal tumors, and others), in addition to abdominal trauma.

The study population was selected following the established inclusion and exclusion criteria. All individuals admitted to a general surgery unit, aged 20 years or older, of both sexes, affected by diseases of the gastrointestinal tract and candidates for surgery, whose nutritional risk screening had been performed within 72 h, participated in the study and underwent anthropometric assessment within 48 h of admission. Individuals hospitalized in other units, under the age of 20 years, admitted in the postoperative period, bedridden individuals, pregnant and lactating individuals, those with severe infection or sepsis, and those with incomplete information or incompatible with the established period, were excluded from this study.

Thus, 331 individuals were included in the study. When calculating the a posteriori sample power (1 − β) for the sample of 331 individuals at a significance level (α) of 0.05 and precision of 2%, a final power to detect malnutrition of 87% was calculated.

Data were collected between August and October 2020, recording demographic information (sex, age), clinical information (length of stay, whether the surgery was oncological or not, percentage of lymphocytes, absolute value of leukocytes, and level of C-reactive protein [CRP]), and nutritional information (weight, height, and nutritional risk screening).

After data collection, the variables were categorized. Subjects were grouped according to age into adults (20-59 years), elderly 1 (60-69 years), and elderly 2 (70 years or older). All individuals were screened using the same Nutritional Risk Screening instrument, the Nutritional Risk Screening (NRS-2002)^15^, standardized by the nutrition service of the hospital in question. Scores were classified as not suggestive of nutritional risk when the sum was less than 3 points, and suggestive of nutritional risk for individuals who scored 3 points or more.

As for the evaluation of the clinical variable immunological depletion, this was performed using the equation of the total lymphocyte count (CTL), equal to the ratio between the percentage of lymphocytes multiplied by the absolute value of leukocytes, divided by one hundred (i.e., CTL = % lymphocytes × leukocytes/100). Findings were interpreted using the following cutoff points (eutrophy: >2,000 cells/m³; mild depletion: 1,200-2,000 cells/m³; moderate depletion: 800-1,199 cells/m³; severe depletion: <800 cells/m³)^3^.

The length of hospital stay was determined using the interval between the date of admission to the unit and hospital discharge, transfer to another unit, or death. It was categorized as ≤15 days or >15 days), according to the median of days of hospitalization.

**Figure 1 -:**
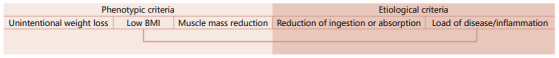
Phenotypic and etiological criteria according to the GLIM and parameters used for the diagnosis of malnutrition among surgical patients at a general hospital in the city of Salvador, Bahia, Brazil, 2021. BMI: body mass index.

Regarding nutritional status, individuals were classified as malnourished or well-nourished. For the diagnosis of malnutrition, the criteria established by the GLIM were adopted, through the association between a phenotype criterion and an etiological criterion, as described in Figure 1.

In verifying the phenotypic criterion, the body mass index (BMI) was calculated by dividing the patient’s weight by their height squared [i.e., BMI = weight (kg)/height² (m)]. For the classification of adult individuals, the cutoff points established by the World Health Organization (WHO) were used (underweight: BMI <18.5 kg/m²; normal weight: BMI 18.5-24.9 kg/m²; overweight: BMI 25.0-29.9 kg/m²; obesity: BMI >30 kg/m²)^30^. As for the classification of elderly individuals, the cutoff points defined by the Pan American Health Organization (PAHO) were used (low weight: BMI <23 kg/m²; adequate weight: BMI 23-28 kg/m²; overweight: BMI 28-30 kg/m²; obesity: BMI >30 kg/m²)^19^.

Among the etiological criteria of the GLIM method, the burden of disease/inflammation was used, based on the adequacy of CRP. The cutoff points were established by Williamson and Snyder^30^, being considered normal when lower than 3 mg/L and increased when 3 mg/L or greater.

For the classification of malnutrition severity, the cutoff points defined by the GLIM based on the BMI phenotypic criterion were used, being either phase 1 or moderate (BMI: <20 kg/m² if <70 years and BMI: <22 kg/m² if >70 years) or stage 2 or severe (BMI: <18.5 kg/m² if <70 years and BMI: <20 kg/m² if >70 years)^4^.

Data were statistically analyzed using SPSS (Statistical Package for the Social Science), version 20.0.0. Categorical variables were presented as absolute and relative frequencies and, after the Shapiro-Wilk normality test, continuous variables were described using medians and interquartile ranges. The validity of the findings was evaluated using the chi-square test for categorical variables and the Mann-Whitney U test for continuous variables. A p-value <0.05 was used to indicate statistical significance for all tests.

Binary logistic regression models (bivariate and multivariate) were used to assess the association between the diagnosis of malnutrition using the GLIM method and clinical (i.e., surgical diagnosis, length of hospital stay, and immunological depletion), and nutritional variables (i.e., NRS-2002 nutritional screening method). Models were adjusted for sex, age, and diabetes diagnosis. A 95% confidence interval (CI) was adopted for the estimated odds ratio (OR).

This study did not involve direct patient exposure, presenting minimal risks of invasion of privacy or unnecessary exposure. The data collected were secondary, meaning that the requirement for free and informed consent of the participants was waived. This study was approved by the Research Ethics Committee of the Universidade do Estado da Bahia, under protocol number 4.222.186, CAAE 35945220.2.0000.0057.

## RESULTS

The main characteristics of the 331 individuals evaluated are shown in Table 1. The sample is predominantly female (57.1%); however, there is a higher percentage of malnutrition cases among men (p=0.008). The median age of study participants was 60 years, being significantly higher among malnourished individuals (67 years).

**Table 1 -:** Demographic, anthropometric, and clinical characterization of surgical patients, according to diagnosis by the GLIM criteria, from a general hospital in the city of Salvador, Bahia, Brazil, 2021.

Characteristics	Total	Well nourished Median (IQ)	Malnourished Median (IQ)	p-value
**Demographics**				
Gender (%)				
Male	142 (42.9)	85 (37.9)	57 (53.3)	0.008*
Female	189 (57.1)	139 (62.1)	50 (46.7)
Age (year)	60 (IQ: 44-70)	55(IQ: 40-67)	67 (IQ: 54-74)	0.000**
**Anthropometric**				
Weight (kg)	61.3 (IQ: 51,9-73,0)	67.8 (IQ: 60.7-77.9)	47.4 (IQ: 43.2-52.8)	0.000**
**Clinics**				
Length of stay (%) (days)				
15	142 (42.9)	100 (44.6)	42 (39.3)	0.354*
>15	189 (57.1)	124 (55.4)	65 (60.7)	
Systemic arterial hypertension (SAH) (%)	125 (37.8)	93 (74.4)	32 (25.6)	0.042*
Diabetes mellitus (DM) (%)	50 (15.1)	37 (74.0)	13 (26.0)	0.299*
Acute kidney injury (AKI) (%)	18 (5.4)	11 (61.1)	7 (38.9)	0.540*
Total lymphocyte count (CTL) (mm³)	1422 (IQ: 1036.2-1908)	1500.9 (IQ: 1129.3-1953.8)	1221.1 (IQ: 845.6-1725)	0.001**
Candidates for oncological surgery (%)	212 (64)	144 (67.9)	68 (32.1)	0.396

Results presented as absolute and relative frequency for categorical variables and median and interquartile range for continuous variables.

IQ: interquartile range.

*Chi-square test.

**Mann-Whitney U test.

The length of hospital stay ranged from 9 to 29 days, and the median was higher (18 days) in malnourished patients compared to well-nourished patients (17 days) (p=0.450).

Median weight and CTL were significantly lower in the group of malnourished individuals in the sample. In addition, among the chronic and acute injuries identified in the study, only systemic arterial hypertension was significantly different in prevalence between the groups.

Table 2 presents the data according to the nutritional risk screening using the NRS-2002, where it can be seen that most of the sample was at risk of malnutrition. As for the diagnosis of malnutrition, based on the association between two criteria established by the GLIM (i.e., low BMI and high CRP), it was found that 32.3% of the individuals admitted to the unit had some degree of malnutrition. Regarding the stratification of malnutrition severity, it can be seen that the frequency of cases of severe malnutrition is higher both among adults (11.5%) and among the elderly (9.7%).

**Table 2 -:** Nutritional risk screening using NRS-2002 and diagnosis of malnutrition based on the GLIM criteria of surgical patients in a general hospital in the city of Salvador, Bahia, Brazil, 2021.

Characteristics	Absolute frequency (N)	Relative frequency (%)
**Nutritional risk (%)**		
<3 - No risk	165	49.8
>3 - At risk	166	50.2
**Malnutrition diagnosis - GLIM (%)**		
Well-nourished	224	67.7
Malnourished	107	32.3
**Severity of malnutrition (%)**		
**Adult**		
Moderate malnutrition	24	7.3
Severe malnutrition	38	11.5
**Elderly**		
Moderate malnutrition	13	3.9
Severe malnutrition	32	9.7

As can be seen in Table 3, the nutritional risk variable identified using the NRS-2002 tool, without adjustment or regardless of the type of adjustment performed, is associated with an increased chance of having a diagnosis of malnutrition using the GLIM method. It is noteworthy that the magnitude of the effect was greater in model 3 (OR=5.8), that is, increased nutritional risk according to the NRS-2002 increases the chances of a diagnosis of malnutrition using the NRS-2002 by 5.8-fold. The GLIM method, in individuals without nutritional risk in the preoperative period, was statistically significant when adjusted for sex, age, diagnosis of diabetes mellitus, type of surgery, immunological depletion, and length of hospital stay.

**Table 3 -:** Binary logistic regression models evaluating the association between malnutrition using the GLIM method and clinical and nutritional variables of surgical patients at a general hospital in Salvador, Bahia, Brazil, 2021.

Variables	OR bivariate (95%CI)	OR multivariate (95%CI)					
		Model 1	Model 2	Model 3	Model 4	Model 5	Model 6
**Nutritional risk (NRS-2002)**	5.833* (3.436-9.903)	4.528* (2.608-7.861)	4.622* (2.649-8.067)	5.791* (3.201-10.478)	-	-	-
**Candidates for oncology surgery**	0.975 (0.604-1.576)	1.310 (0.783-2.182)	1.266 (0.757-2.108)	-	2.068** (1.161-3.683)	-	-
**Immune depletion**	1.571 (0.869-2.839)	1.125 (0.600-2.109)	1.085 (0.576-2.043)	-	-	1.494 (0.770-2.898)	-
**Hospitalization time**	1.007 (0.995-1.020)	1.006 (0.993-1.019)	1.005 (0.992-1.019)	-	-	-	1.010 (0.996-1.024)

OR: odds ratio; CI: confidence interval.

*p<0.001; **p<0.05.

Reference categories: malnutrition, presence of nutritional risk, oncological type surgery and presence of immunological depletion.

Model 1: Adjusted for age and sex.

Model 2: Adjusted for age, sex, and diabetes mellitus diagnosis.

Model 3: Adjusted for age, sex, diagnosis of diabetes mellitus, oncologic surgery, immune depletion, and length of hospital stay.

Model 4: Adjusted for age, sex, diagnosis of diabetes mellitus, nutritional risk, immune depletion, and length of hospital stay.

Model 5: Adjusted for age, sex, diagnosis of diabetes mellitus, nutritional risk, oncologic surgery, and length of stay.

Model 6: Adjusted for age, sex, diagnosis of diabetes mellitus, oncologic surgery, and immune depletion.

Patients who were the candidates for oncologic surgery were twice as likely to have malnutrition according to the GLIM, after adjusting the analysis for sex, age, diagnosis of diabetes mellitus, nutritional risk, immunological depletion, and length of hospital stay (p=0.014).

Although a tendency was observed for immunological depletion and length of hospital stay to increase the chances of malnutrition diagnosis using the GLIM method, the results were not statistically significant.

## DISCUSSION

This study identified a high prevalence of nutritional risk using the NRS-2002 tool and malnutrition using the GLIM method in surgical patients at the largest public hospital in the northeast region of Brazil, highlighting that severe malnutrition was predominant regardless of age group. In addition, malnutrition was found to be associated with nutritional risk as detected using the NRS-2002 nutritional screening tool and with oncologic surgery, after statistical adjustment.

It is widely accepted that malnutrition negatively affects patient outcomes, as preoperative nutritional status is an important determinant of postoperative outcomes^6^ ^,^ ^24^. Patients who are malnourished at the time of surgery are almost 30% more likely to develop serious surgical complications and twice as likely to die in the 30 days after surgery compared to well-nourished patients^22^.

In this study, it was found that almost one-third of the patients in the preoperative period (32.3%) were diagnosed with some degree of malnutrition as they met the combination of the adopted GLIM criteria (i.e., low BMI and inflammation). This prevalence was higher than that described by Henrique et al.^13^ when using the same combination of criteria (20.4%) and also higher than the finding by Steer et al.^23^ (22.6%), when adopting low BMI, percentage of weight loss, reduction of muscle mass, and reduction of food intake in association with the presence of metastatic disease. However, it was lower than that described by Laty et al. ^16^, who classified 46.9% of patients as malnourished using the GLIM criteria, but did not specify which criteria were adopted.

Similar to this study, an observational and cross-sectional survey carried out with cancer patients in Australia detected a prevalence of malnutrition similar to that found (using an unspecified combination of GLIM criteria), showing that 35% of patients were identified as malnourished^7^. Another finding, also similar to the one reported, was made in a retrospective Norwegian study, comprising 6,110 surgical patients, in which 35.4% of the patients were malnourished at the time of surgery, according to the GLIM criteria, using the criteria of reduced nutrient absorption, BMI, and weight loss^22^.

It should be noted that in the sample evaluated, abdominal surgeries predominated, whether oncological or not, since the patients were affected by diseases in the gastrointestinal tract, explaining the relative prevalence of malnutrition. Weight loss and malnutrition are believed to be prevalent among individuals with gastrointestinal, hepatobiliary, and pancreatic diseases, as a result of the involvement of the digestive organs and their metabolic functions. However, both the frequency and severity of malnutrition in these patients are not well-described in the literature^22^ ^,^ ^11^.

Nutritional risk is possibly reversible, so its early recognition can play an important role in preventing the development of malnutrition and improving clinical outcomes^24^. In this study, 50.2% of the patients were at risk of malnutrition according to the NRS-2002. Other studies carried out with adult patients affected by diseases of the gastrointestinal tract and patients admitted to a general surgery unit identified findings that ranged from 17.1%^11^ to 62.2%^26^ in the prevalence of nutritional risk according to the NRS-2002. Sun et al.^24^ reported that this is a reliable tool that is easy to apply and capable of identifying patients at nutritional risk.

A meta-analysis of 3,527 patients undergoing abdominal surgery in Asia and Europe showed a strong correlation between preoperative nutritional risk and increased rates of complications, mortality, and length of hospital stay^24^. This last finding was also described by Garcia et al^10^.

Patients identified to be at nutritional risk in this study were more likely to be diagnosed with malnutrition using the GLIM criteria. A study carried out in Romania with 3,198 adult patients, admitted to the gastroenterology sector, identified that patients with nutritional risk according to the NRS-2002 and cancer patients are more prone to the development of malnutrition^11^.

It is estimated that about 30-90% of cancer patients suffer from malnutrition ^7^. In this study, it was seen that most of the individuals (64%) had a surgical diagnosis related to oncological disease in their clinical history. These candidates for oncological surgery were twice as likely to develop malnutrition according to the GLIM (after statistical adjustment), which corroborates the finding by Williams et al^28^ , who found that two-thirds of patients who are the candidates for oncological and gastrointestinal surgeries are malnourished in the preoperative period.

It is indisputable that cancer negatively affects the nutritional status of individuals, since it triggers enormous changes and metabolic responses to the state of persistent inflammation^9^. Malnutrition in cancer can be caused by numerous factors, such as the location of the tumor, the organs involved, the patient’s response, and the treatment used^20^. Thus, it is believed that patients with head and neck cancer or malignant tumors in the digestive tract are at greater risk of developing malnutrition than patients with other types of cancer^12^.

Studies show that the immune system is intensely affected by malnutrition, sometimes presenting an insufficient response to bacteria, viruses, and fungi. About 20.5% of malnourished patients express low levels of CTL^2^ ^,^ ^27^, and a study by Rocha and Fortes^21^ points to CTL levels as a predictor of risk of postoperative complications. Although immunological depletion is not a risk factor for malnutrition in the evaluated sample, the CTL was significantly lower in the group of malnourished individuals, suggesting lower immune reserves and, consequently, a deficit in defense mechanisms.

Finally, there was no statistically significant association between length of stay and diagnosis of malnutrition using the GLIM method in this study. However, the median number of days of hospitalization was high when compared to other studies carried out with surgical patients, which can be explained by the high demand for surgeries at the reference hospital in general surgery where the study was carried out^1^ ^,^ ^14^.

There are still few studies validating the use of the GLIM criteria and their application to surgical patients. Furthermore, depending on the combination of the phenotype and etiological criteria used, different rates of malnutrition prevalence can be verified in the same population^16^ ^,^ ^13^. However, this fact does not invalidate the importance of this methodology, which seeks to unify the diagnostic criteria for malnutrition based on a robust assessment of each individual, involving a combination of clinical, biochemical, and anthropometric parameters^4^.

Among the limitations found, we highlight the fact that this was a retrospective study where the available data did not cover all the criteria listed by the GLIM for a broader and comparative analysis with other studies. In addition, long-term outcomes such as late complications, readmissions, or deaths were not evaluated. On the other hand, the strengths of this study include that data collection was performed by a single professional, in a standardized way, and the study population included only surgical patients, predominantly with diseases of the gastrointestinal tract. In addition, this study can contribute to the data on malnutrition according to the GLIM criteria in this population, information that is still scarce.

## CONCLUSION

This study identified a high prevalence of nutritional risk using the NRS-2002 tool and malnutrition using the GLIM method in surgical patients, highlighting the predominance of severe malnutrition among the evaluated individuals. In addition, preoperative patients with nutritional risk, as detected using the NRS-2002 nutritional screening tool, and candidates for oncologic surgery are more likely to be diagnosed as malnourished using the GLIM criteria.

It is, therefore, recommended that further studies be carried out in surgical patients, reinforcing the use of this new robust method of diagnosing malnutrition, in order to promote more effective nutritional interventions and contribute to the reduction of morbidity and mortality, and hospital costs in this population.
